# Extensions of the mTPI and TEQR designs to include non-monotone efficacy in addition to toxicity for optimal dose determination for early phase immunotherapy oncology trials

**DOI:** 10.1016/j.conctc.2018.01.006

**Published:** 2018-01-31

**Authors:** Revathi Ananthakrishnan, Stephanie Green, Daniel Li, Michael LaValley

**Affiliations:** a Department of Biostatistics, Boston University, 801 Massachusetts Avenue 3rd Floor, Boston, MA 02118, USA; b New London, CT 06320, USA; c Juno Therapeutics, Seattle, WA, USA

**Keywords:** Early phase immunooncology design considering efficacy and safety, Extended mTPI design, Extended TEQR design, Optimal biological dose isotonic design, Eff-Tox design, Umbrella-shaped dose-response curve

## Abstract

With the emergence of immunotherapy and other novel therapies, the traditional assumption that the efficacy of the study drug increases monotonically with dose levels is not always true. Therefore, dose-finding methods evaluating only toxicity data may not be adequate. In this paper, we have first compared the Modified Toxicity Probability Interval (mTPI) and Toxicity Equivalence Range (TEQR) dose-finding oncology designs for safety with identical stopping rules; we have then extended both designs to include efficacy in addition to safety – we determine the optimal dose for safety and efficacy using these designs by applying isotonic regression to the observed toxicity and efficacy rates, once the early phase trial is completed. We consider multiple types of underlying dose response curves, i.e., monotonically increasing, plateau, or umbrella-shaped. We conduct simulation studies to investigate the operating characteristics of the two proposed designs and compare them to existing designs. We found that the extended mTPI design selects the optimal dose for safety and efficacy more accurately than the other designs for most of the scenarios considered.

## 1 Introduction

Several dose finding oncology designs have been developed that are improvements over the 3 + 3 design in terms of accuracy of maximum tolerated dose (MTD) selection as well as other operating characteristics such as the percentage of patients under-dosed [[1], [2], [3], [4], [5], [6]]. There are also designs that incorporate efficacy in dose selection, in addition to safety. These include the seamless Phase 1/2 SEARS design [7,8], a seamless 2-step Phase 1/2 design [9,10], designs to find the optimal biological dose [11,12], the Eff-Tox design [13,14] and the Toxicity and Efficacy Probability Interval (TEPI) design [15] among others [[16], [17], [18], [19]]. In this paper, we focus on two relatively recent dose-finding designs that have been proposed to determine the MTD, namely the mTPI and the TEQR designs [4,5], and then extend them to choose the optimal dose for both safety and efficacy. Our aim is to identify the best (optimal) dose using a practical design, and not specifically to optimize our proposed design(s). We define the optimal dose to be the dose with the highest efficacy below or at the MTD. The mTPI design is a Bayesian dose finding design, where the dose finding decisions are based on whether a statistic called the Unit Probability Mass (UPM) has its highest value in the target dose limiting toxicity (DLT) interval or in the interval above or below it. The TEQR design uses a similar concept for dosing decisions but provides a frequentist counterpart to the Bayesian mTPI design, since the dosing decisions in the TEQR design are based on the empirical DLT rates.

Phase I trials are generally very small and the accuracy of MTD selection is low with such a small sample size. Hence, we first compare the frequentist TEQR and the Bayesian mTPI dose-finding designs for accuracy of MTD selection for various sample sizes while requiring identical stopping rules. We then extend the mTPI and TEQR designs with a moderately large sample size to choose an optimal dose based on both safety and efficacy by considering safety and efficacy outcomes using Bernoulli distributions.

A key part of our evaluation of these designs is to determine their performance when the efficacy response rate does not necessarily increase monotonically with increasing dose. With immunotherapy and other novel therapeutics, the traditional assumption of increasing efficacy with increasing dose may no longer hold [11,20]. Thus, in our simulations to evaluate these designs, we assume that the true DLT rates increase monotonically with an increase in dose but we do not assume that this is true for the efficacy response rates. We allow multiple types of curves for dose-response in the simulations: monotonically increasing, plateau, or umbrella-shaped curves. In this context of potentially non-monotone efficacy, we apply isotonic regression to the differences in observed response rates between adjacent dose levels and investigate its use in selecting an optimal dose for safety and efficacy.

The work by Li et al. [15] proposes using a statistic called the joint unit probability mass (JUPM) to incorporate both toxicity and efficacy, to extend the mTPI design. This Toxicity and Efficacy Probability Interval (TEPI) design, as well as other designs such as the Eff-Tox design and the Optimal Biological Dose (OBD) Isotonic design [12], requires that efficacy or a surrogate of efficacy be available in a similar time frame as the DLT observation period, for dosing decisions. Our extended mTPI and TEQR designs do not require this, since we use the efficacy information for optimal dose selection only at the end of the trial. Thus, we propose a simple way of extending the mTPI and TEQR designs to include efficacy in dose selection, using isotonic regression. We finally compare the accuracy of dose selection of the extended mTPI and TEQR designs to that of the Eff-Tox design, the OBD Isotonic design and the TEPI design.

## 2 Methods

### 2.1 mTPI and TEQR designs

The mTPI design is a Bayesian design that uses the unit probability mass (UPM) statistic, defined as the ratio of the probability mass of the interval and the length of the interval [4], for the dose finding decisions. The toxicity probability scale is divided into three intervals, namely (0, p_T_-ε_1_), [p_T_-ε_1_, p_T_+ε_2_] and (p_T_+ε_2_, 1), where p_T_ is the target probability of DLT and ε_1_ and ε_2_ are used to define the interval for the target DLT rate. These three intervals correspond to under-dosing, correct dosing and over-dosing respectively. The rules for escalating, staying at the same dose or de-escalating depend on which of these intervals has the highest UPM for that dose level, based on a beta-binomial posterior distribution formed from the likelihood of the observed DLT data and a beta (1,1) prior. For example, the next cohort of patients will be treated at the same dose if the UPM is the largest for the correct dosing interval. The trial stops if dose level 1 is too toxic or if the pre-specified maximum sample size is reached or exceeded.

The TEQR design is a frequentist design based on the empirical DLT rate [5]. As in the mTPI design, the toxicity probability scale is divided into three intervals, namely (0, p_T_-ε_1_), [p_T_-ε_1_, p_T_+ε_2_] and (p_T_+ε_2_,1). The rules for escalating, staying at the same dose or de-escalating depend on which of these intervals contains the empirical DLT rate for that dose level – for example, if the empirical DLT rate lies in the interval [p_T_-ε_1_, p_T_+ε_2_], the next cohort of patients will be treated at the same dose. The trial stops if dose level 1 is too toxic or when a dose level achieves the pre-specified MTD sample size. In both the mTPI and TEQR design, we stay at the current dose if the current dose is safe but the DLT data indicate that the next higher dose is too toxic.

### 2.2 Using isotonic regression on DLT rates and on monotonically increasing or plateauing response rates to determine the optimal dose

When the true underlying DLT rate (or response rate) increases with an increase in dose, the observed DLT (or response) rate is also expected to be a monotonically non-decreasing function of dose. However, this may not always be what is observed due to the small sample size in each dose level in dose-finding oncology trials. Isotonic regression is a weighted regression and a smoothing procedure that can be used to provide estimates of the DLT (or response) rate that are monotonically non-decreasing functions of dose [21]. This then enables us to determine the highest dose level that is acceptable for safety and the lowest dose level that is acceptable for efficacy.

**Fig. 1:**
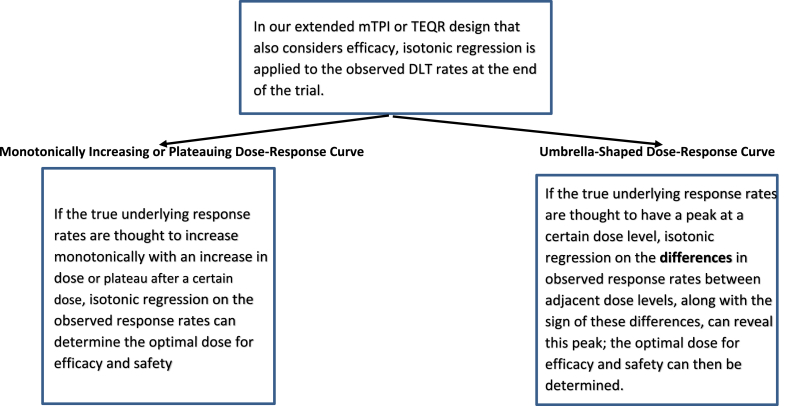
Schematic of analysis method for different dose-response curves.

In a trial using a standard mTPI or TEQR design, the dose chosen for safety is the highest dose level with a DLT rate that is closest to (and below) the pre-specified DLT threshold rate (say 0.33) after applying isotonic regression at the end of the trial to the observed DLT rates. In our extensions of the mTPI and TEQR designs, isotonic regression is also applied independently to the observed efficacy response rates at the end of each trial, when the true underlying response rates are thought to be monotonically increasing or monotonically non-decreasing with an increase in dose. Since the estimated response rates will be monotonically non-decreasing with an increase in dose after applying isotonic regression, we choose as the optimal dose for safety and efficacy the highest dose level where the DLT rate is less than or equal to 0.33 after isotonic regression, only if the smoothed response rate at that dose level is equal to or above the efficacy threshold (say response rate of 0.4). For example, if dose level 4 is chosen after isotonic regression as the highest dose level with a DLT rate <= 0.33 and dose level 3 or lower is chosen after isotonic regression as the lowest dose level with a response rate >= 0.4, then dose level 4 is the optimal dose for safety and efficacy since the response rate at dose level 4 will be >= 0.4 in this monotone case. However, if dose level 3 is chosen for safety after isotonic regression and dose level 4 is chosen for efficacy after isotonic regression, then no dose level is optimal for safety and efficacy because the efficacy threshold of a response rate of 0.4 is not crossed at dose level 3, but only at dose level 4. If dose level 3 is chosen for both safety and efficacy after isotonic regression, then dose level 3 is the optimal dose for safety and efficacy (Fig. 1, Fig. 3).

**Fig. 2:**
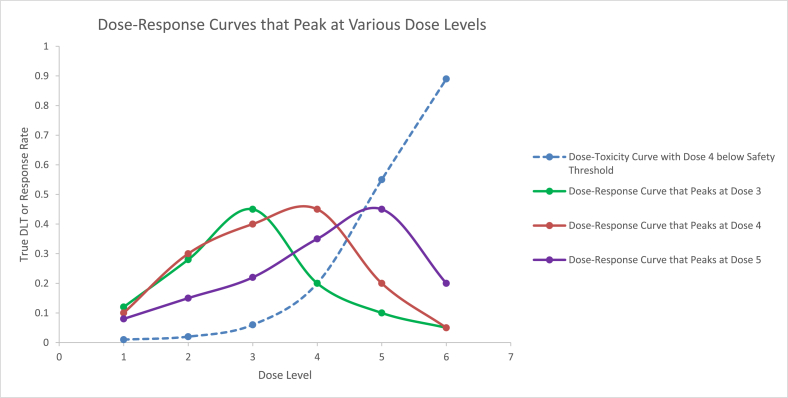
In this example, Dose level 4 is below the toxicity threshold rate of 0.33 (blue curve with dashes). For the green dose-response curve with the peak response rate at dose level 3, dose level 3 is chosen as the optimal dose for toxicity and efficacy, assuming the peak response rate is above the efficacy threshold at dose level 3. For the brown dose-response curve with the peak response rate at dose level 4, dose level 4 is chosen as the optimal dose, assuming the peak response rate is above the efficacy threshold at dose level 4. For the purple dose-response curve with the peak response rate at dose level 5, dose level 4 is chosen as the optimal dose, only if the response rate at dose level 4 reaches the efficacy threshold – if not, no dose is chosen as the optimal dose.

**Fig. 3:**
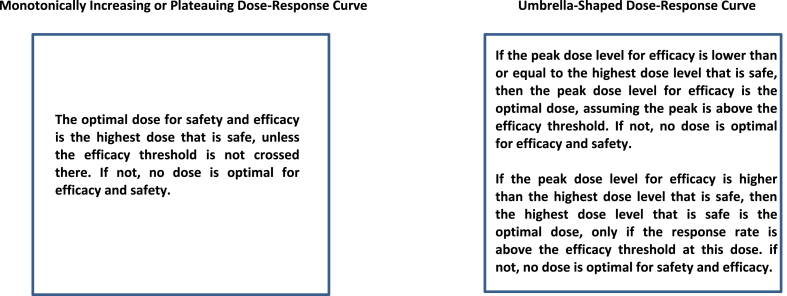
Summary of optimal dose selection for various dose-response curves.

### 2.3 Finding the peak of an umbrella-shaped dose response curve using isotonic regression

The OBD Isotonic design by Zang et al. [12] uses a double-sided isotonic approach to determine the peak of an umbrella-shaped dose response curve. We use a simpler method to determine the peak. When there is a peak in the dose-response curve, an umbrella-shaped dose-response curve, we apply isotonic regression to the differences in observed response rates between adjacent dose levels obtained at the end of each simulated trial. These differences provide the change between discrete dose levels and function like a derivative, or rate of change. For a convex curve, the derivative is 0 at the peak, and the sign of the derivative changes from positive before the peak to negative after the peak. This provides the approach we use to determine the peak of an umbrella shaped dose-response curve – we apply isotonic regression at the end of each simulated trial to the differences in observed response rates between adjacent dose levels. As the version of isotonic regression we used allows only monotonically non-decreasing estimates, the differences were constructed to be negative when the curve increases and positive when the curve decreases. Applying isotonic regression to the differences, we observe where the sign of the differences switches from negative to positive, to determine the peak of the curve (Fig. 1). This method to determine the peak of an umbrella-shaped dose response curve is demonstrated to work well with examples in the Results Section and Appendix Section 5. Once the peak of the dose response curve is found, the optimal dose for efficacy and safety can be determined, as explained in Fig. 2, Fig. 3.

### 2.4 Comparisons of results for accuracy of optimal dose selection

We compare the results for accuracy of optimal dose selection of the extended mTPI and TEQR designs to those of the Eff-Tox, OBD Isotonic and TEPI designs for various scenarios of true toxicity and efficacy rates. The Eff-Tox design is a Bayesian design that considers the trade-off between the probabilities of drug toxicity and efficacy to determine the optimal dose for each new cohort of patients. The stopping point of the trial is usually at a pre-specified sample size. Further details are provided in the references by Thall et al. [13,14]. The details of the OBD Isotonic design are provided in the reference by Zang et al. [12]. To determine the OBD, an admissible set of doses satisfying a safety criterion similar to that used in the Eff-Tox design, is first defined. The OBD is then the lowest dose with the highest response rate within the admissible set of doses, while still being safe. The stopping point of the trial is usually at a pre-specified sample size. As mentioned earlier, the TEPI design is an extension of the mTPI design that includes efficacy and safety in dose selection. The stopping point of the trial is usually at a pre-specified sample size. Further details are provided in the reference by Li et al. [15].

### 2.5 Simulation structures

We generate two Bernoulli distributed binary random variables for the toxicity and efficacy outcomes of simulated patients – these random variables can be generated as either uncorrelated or correlated. In most of the simulations presented in this paper, we generate the DLT occurrence for patients at each dose level from values based on a logistic dose toxicity curve, whose two coefficients are calculated using the following parameters: true DLT rate at starting dose (dose level 1, 100 units) of 0.01 and true DLT rate of 0.2 at the MTD (dose level 4, 501 units). However, the dose response curve for the efficacy of simulated patients at each dose level varies by the simulation scenario, with 3 possibilities: it can monotonically increase, increase until reaching a plateau and then remain at the same level, or follow an umbrella-shape where it increases until reaching a peak after which it decreases (Table 1).

**Table 1:** Monotonically increasing true DLT rates with an increase in dose and different dose-response curves.

Dose	Dose Level	Probability of DLT^a^	Monotonically Increasing True Response Rates	Plateauing Response Rates with an Increase in Dose	Umbrella-Shaped Dose-Response Curve
100 units	1	0.01	0.1	0.1	0.1
200	2	0.02	0.3	0.3	0.35
334	3	0.06	0.4	0.4	0.5
501	4	0.2	0.45	0.45	0.3
701.4	5	0.55	0.55	0.45	0.2
932.86	6	0.89	0.6	0.45	0.05

a True probability of DLT at each dose, generated from a logistic curve, whose coefficients are calculated assuming the probability at a dose of 100 units to be 0.01 and at 501 units to be 0.2. The dose levels follow the modified Fibonacci series. Log_e_ (DLT rate/(1-DLT rate)) = -5.39533 + 0.008002 × dose.

We have created SAS codes, available on request, to simulate both the extended mTPI and TEQR designs. To obtain the statistical operating characteristics for each design, we perform 1000 simulated trials for each scenario. The rules for escalation, de-escalation or remaining at the same dose for each simulated trial are based on the number of observed DLTs. Two different stopping rules are considered in our simulations, which are the usual stopping rules for the mTPI and TEQR designs respectively; a simulated trial stops when a) the total planned sample size is reached or b) the planned MTD sample size is reached. For both stopping rules, the simulated trial would also stop if dose level 1 is determined to be too toxic.^1^ In our simulations of these designs, we also track the efficacy response of each patient and the resultant efficacy response rate at each dose level. Although the dose escalation/staying/de-escalation decisions during the trial are determined only by the number of observed DLTs, at the end of each simulated trial we choose a dose that is optimal for both safety and efficacy based on the observed DLT and response rates at each dose level (Fig. 1, Fig. 2, Fig. 3).

The input parameters used in our SAS code for the mTPI and TEQR designs are provided in Appendix Table 1. The coefficient of correlation r between efficacy and toxicity is set to 0 (independent true toxicity and efficacy rates) for the simulation results presented in the main text. This is because Cai and co-authors [22] showed that joint modeling of efficacy and safety does not necessarily improve the performance of the dose finding, especially when efficacy is weakly correlated with toxicity. However, the results can be investigated for correlation coefficients other than zero (Appendix Section 2) within the valid range of values that the correlation coefficient can assume.

The simulations presented in this paper consider the following scenarios: 1) as a reference, we consider only toxicity rates and ignore efficacy; 2) both toxicity and response rates increase with increasing dose; 3) toxicity rates increase with increasing dose, and response rates are monotonically increasing but reach a plateau after a certain dose; 4) toxicity rates increase with increasing dose, but the response rate has an umbrella-shape with a peak at an intermediate dose.

We then compared the accuracy of dose selection of the extended mTPI and TEQR designs with that of the Eff-Tox, OBD Isotonic and TEPI design. These simulations comparing the various designs include the scenarios above (monotonically increasing, plateauing and umbrella-shaped dose-response curves).

## 3 Results

### 3.1 mTPI and TEQR designs: safety only

Only the monotonically increasing DLT rates with increasing dose shown in Table 1 are used in the simulations for Table 2 with no efficacy considered; isotonic regression is applied to the observed DLT rates at the end of each simulated trial to determine the MTD.

**Table 2:** Results for Accuracy of MTD Selection Using the Stopping Rules of the mTPI Design^b^.

Total Sample Size	Cohort Size	mTPI Accuracy of MTD Selection^a^	% of Patients at MTD	% of patients under-dosed	% of patients over-dosed	TEQR Accuracy of MTD Selection^a^	% of patients at MTD	% of patients under-dosed	% of patients over-dosed
40	4	80.3%	53.8%	37.7%	8.5%	68.7%	47.6%	44.1%	8.3%
50	5	86.2%	58.1%	33.3%	8.7%	64.5%	44.6%	48.9%	6.5%
100	10	91.5%	65.7%	28.4%	5.9%	82.8%	56.1%	37.6%	6.3%

60	2	78.1%	65.2%	27.8%	7%	44.1%	39.6%	57%	3.4%
60	3	76.0%	62.9%	31%	6.1%	74.7%	54.4%	39.5%	6.1%
60	4	85.1%	64.1%	29.2%	6.8%	67.2%	52.3%	41.5%	6.2%
60	5	86.4%	62.4%	30.3%	7.3%	66.6%	47.4%	46.6%	5.9%
60	6	82.7%	57.4%	36.7%	5.9%	81.3%	51.2%	42.7%	6.1%
60	10	90.4%	51.5%	40.8%	7.8%	79.7%	43.2%	48.1%	8.7%

18	3	63.8%	37.5%	49.8%	12.7%	50.9%	32.5%	59.1%	8.4%
24	3	69.1%	44.5%	42.7%	12.8%	60.0%	37.8%	52.6%	9.6%
30	3	71.5%	49.5%	39.1%	11.4%	66.6%	42.8%	47.3%	9.9%
36	3	75.2%	54.4%	35.3%	10.4%	68.8%	46.2%	45.2%	8.7%
42	3	76.5%	58%	32.4%	9.6%	71.1%	48.7%	43.2%	8.1%
51	3	77.0%	61%	31.3%	7.7%	73.0%	52.6%	40.9%	6.5%

a % of Times out of 1000 Simulations that Dose Level 4 is Selected as the MTD.

b Trial stops when the total planned sample size is reached or dose level 1 is too toxic (stopping rule a)).

We use the same stopping rules for the mTPI and TEQR designs and compare them for accuracy of MTD selection. We use the usual stopping rules of the mTPI design (stopping rule a)), namely stop the trial when the total planned sample size is reached or when dose level 1 is too toxic, for both the mTPI and TEQR designs and compare their performance for the accuracy of MTD selection (Table 2); dose level 4 with a true DLT rate of 0.2 is the true MTD in this scenario.

Results for the accuracy of MTD selection for the mTPI and TEQR designs when the stopping rules of the TEQR design (stopping rule b)) are used are not shown here. However, in general, when identical stopping rules are used for both the designs, the Bayesian mTPI design is more accurate than the frequentist TEQR design in selecting the true MTD, with the same (Table 2) or a similar number of subjects. Using the UPM statistic for dose finding as in the mTPI design, rather than the empirical DLT rate as in the TEQR design, appears to estimate the MTD more accurately, put a larger percentage of patients at the MTD as well as under-dose a smaller percentage of patients. Although the association between accuracy of MTD selection and cohort size given the same sample size may not be very clear from Table 2, very small or very large cohort sizes would not be optimal. However, it is clear that given the same cohort size, the accuracy of MTD selection increases when the total sample size is increased. Thus, in the following sets of simulations, we use a moderately large sample size of 50 subjects to evaluate efficacy and safety. We show results in the following sections for a cohort size of 5, that is moderate, with a sample size of 50 but our codes can be used to obtain results for other cohort sizes (for e.g. cohort size of 3 with a total sample size of 51).

### 3.2 Extended mTPI and TEQR designs: incorporating safety and efficacy

a) We use the stopping rules of the mTPI design (stopping rule a)), namely stop the trial when the total planned sample size is reached or when dose level 1 is too toxic, for the extended mTPI and TEQR designs for Scenarios 1–3 below and compare their performance for dose selection. The results in Table 3 are based on a total sample size of 50 and a cohort size of 5.

**Table 3:**
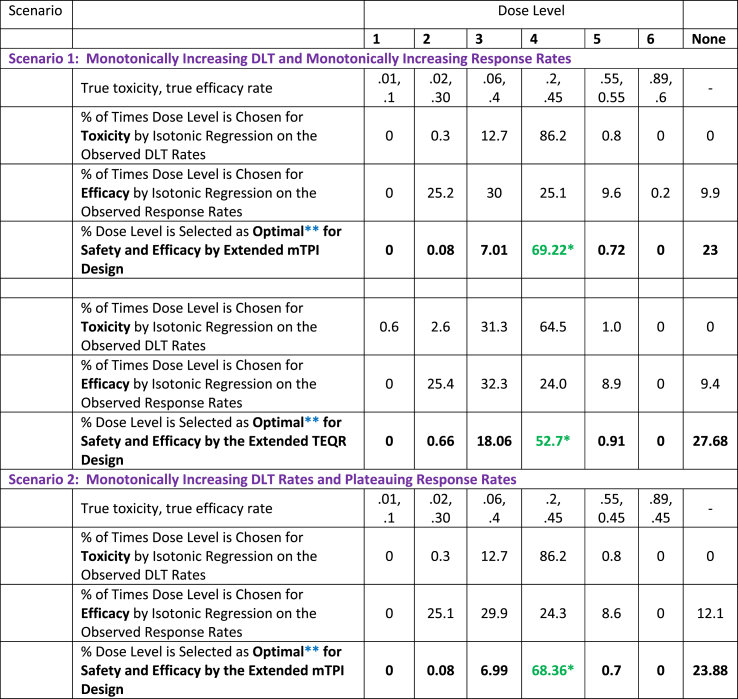

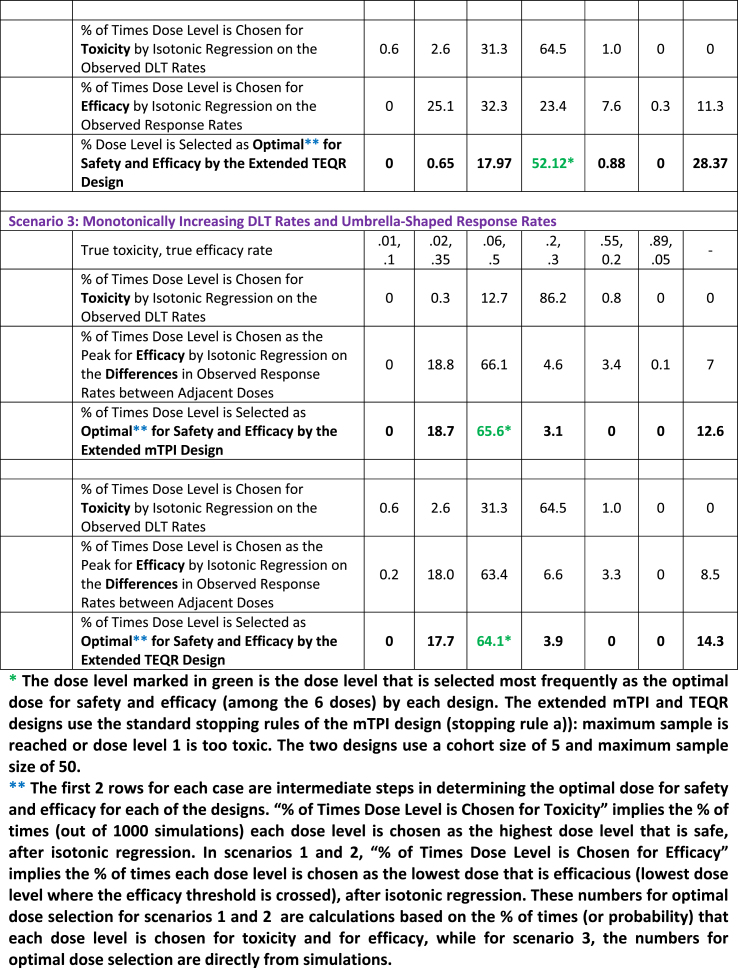
Percentage of Times Each Dose is Selected as Optimal for Safety and Efficacy for the Extended mTPI and TEQR Designs for Three Different Dose Response Curves.
**Scenario 1)** Monotonically Increasing True DLT Rates and Monotonically Increasing True Response Rates with an Increase in Dose
The monotonically increasing true DLT and response rates with an increase in dose shown in Table 1 are used in the simulations and isotonic regression is applied independently to the observed DLT rates and to the observed response rates at the end of each simulated trial.
The results are shown in Table 3. For the monotonically increasing DLT and response rates in Table 1, both the extended mTPI and TEQR designs select dose level 4 as the optimal dose for safety and efficacy with the highest frequency/probability (Table 3). The extended mTPI design selects dose level 4 as the optimal dose with a higher probability than the extended TEQR design does.
**Scenario 2)** Monotonically Increasing True DLT Rates and Plateauing True Response Rates with an Increase in Dose
The monotonically increasing true DLT rates and plateauing response rates with an increase in dose shown in Table 1 are used in the simulations and isotonic regression is applied independently to the observed DLT rates and to the observed response rates at the end of each simulated trial.
The results are shown in Table 3. For the monotonically increasing DLT rates and the plateauing response rates in Table 1, both the extended mTPI and TEQR designs select dose level 4 as the optimal dose for safety and efficacy with the highest frequency/probability (Table 3). The extended mTPI design selects dose level 4 as the optimal dose with a higher probability than the extended TEQR design does. The results in Table 3 for the percentages of dose selection for the plateauing response rates in Table 1 are very similar to those shown in Table 3 for the monotonically increasing response rates in Table 1.
**Scenario 3)** Monotonically Increasing True DLT Rates and True Response Rates that Follow an Umbrella-Shaped Curve
The monotonically increasing true DLT rates with an increase in dose and the umbrella-shaped true response rates shown in Table 1, where the response rate peaks at dose level 3, are used in the simulations; isotonic regression is applied to the observed DLT rates and isotonic regression is applied to the **differences** in observed response rates between adjacent dose levels at the end of each simulated trial.
The results are shown in Table 3. When the true dose-response curve is thought to possess a clear peak, we suggest applying isotonic regression to the **differences** in observed response rates between adjacent dose levels to identify this peak dose level for efficacy, as described in Appendix Section 5 with further examples. The results in Table 3 show that dose level 3 is chosen as the peak for efficacy (and the optimal dose for safety and efficacy) most frequently for both the extended mTPI and TEQR designs, consistent with the peak at dose level 3 in the true underlying response rates shown in Table 1. The extended mTPI design selects dose level 3 as the optimal dose with a higher probability than the extended TEQR design does. Fig. 2, Fig. 3 explain how the optimal dose is selected at the end of each simulation for a dose-response curve with a peak.

b) We also use the usual stopping rules of the TEQR design (stopping rule b)), namely stop the trial when the planned MTD sample size is reached or when dose level 1 is too toxic, for the extended mTPI and TEQR designs for Scenarios 1–3 and compare their performance for dose selection. Isotonic regression is applied to the observed DLT rates, and isotonic regression is applied to the observed response rates (monotonically increasing and plateauing response rates) and to the differences in the observed response rates between adjacent dose levels (umbrella-shaped response rates) at the end of each simulated trial. The simulations are based on a MTD sample size of 50 and a cohort size of 5.
A table of results for dose selection similar to Table 3 is not shown for the 3 scenarios of monotonically increasing, plateauing and umbrella-shaped response rates in Table 1, using the stopping rules of the TEQR design (stopping rule b)). However, the percentages for dose selection for each of the 3 scenarios and each of the designs (extended TEQR and extended mTPI design) are similar to the percentages shown in Table 3. The results are described briefly below.
For the monotonically increasing DLT and monotonically increasing response rates (Table 1), the extended mTPI design and the extended TEQR design select dose level 4 as optimal for safety and efficacy 70% and 53% of the time respectively.
For the monotonically increasing DLT rates and plateauing response rates (Table 1), the extended mTPI design and the extended TEQR design select dose level 4 as optimal for safety and efficacy 70% and 52% of the time respectively.
For the monotonically increasing DLT rates and umbrella-shaped response rates (Table 1), the extended mTPI design and the extended TEQR design select dose level 3 as optimal for safety and efficacy 66.3% and 62.7% of the time respectively.
For the extended TEQR design, we apply isotonic regression to the differences in the observed response rates between adjacent dose levels at the end of each simulated trial; we investigate the properties of this technique in determining the dose for efficacy when the response rates are not umbrella shaped i.e. for monotonically increasing response rates (Appendix Section 3) or plateauing response rates (Appendix Section 4). In both cases, the technique does not work and no dose level is selected as the optimal dose most frequently (Appendix Section 3, Appendix Section 4). For the plateauing response rates, the dose level at which the response rate starts plateauing is not chosen frequently as the peak dose (Appendix Section 4). Thus, applying this technique of isotonic regression to the differences in the observed response rates between adjacent dose levels is not useful in the case when there is no clear peak in the true underlying response rates, but can work well when there is a clear peak in the underlying dose-response curve (Appendix Section 5).

### 3.3 Comparison of the accuracy of optimal dose selection for Various Designs

We compare in Table 4 the accuracy of optimal dose selection of our extended mTPI and TEQR designs to that of the Eff-Tox design, the OBD Isotonic design and the TEPI design, for some scenarios of true DLT and response rates. All the input parameters used in the Eff-Tox design, OBD Isotonic design and TEPI design simulations are provided in Appendix Section 6.

**Table 4:**
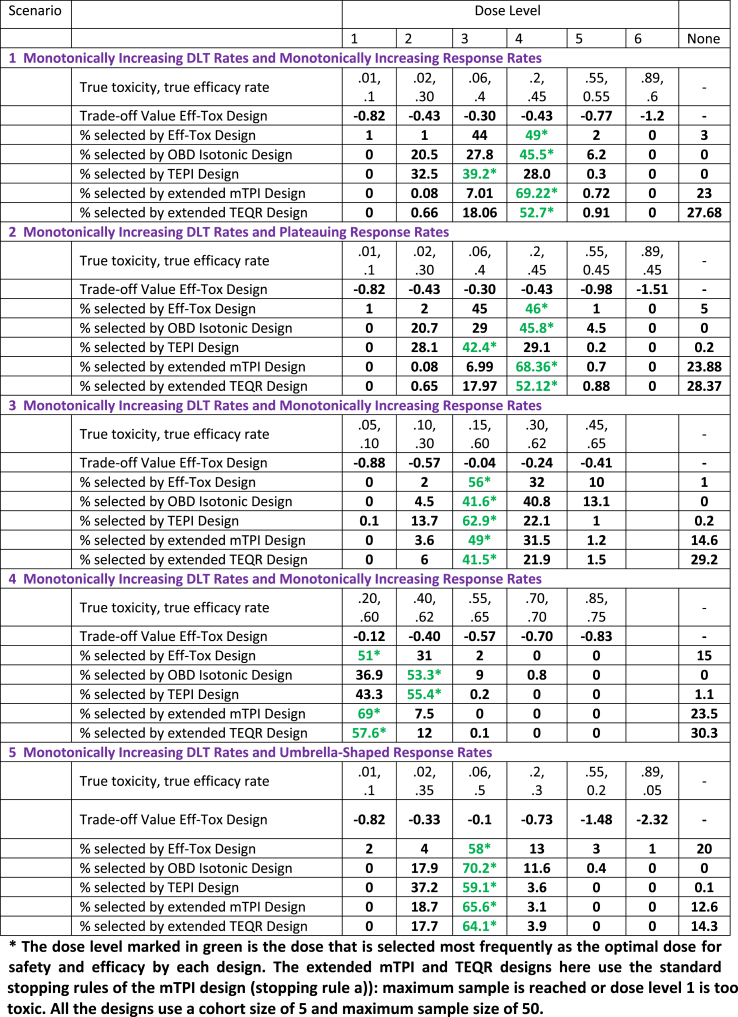
Results for Accuracy of optimal dose selection for various Designs.

For scenarios 1 and 2, where the DLT rate increases substantially between dose levels 3 and 4, while the response rate increases only slightly, the proposed extended mTPI and TEQR designs outperform the other designs with a higher percent of selecting dose level 4 as the optimal dose. The TEPI design has a higher percentage of selecting dose level 3 as the optimal dose, while the other designs pick dose level 4. This may be justified as clinicians may have different judgements on which of these two dose levels is optimal in terms of a trade-off between efficacy and toxicity. The TEPI design selects the dose based on a utility function with a safety and efficacy trade off. In these two scenarios, the utility values for dose levels 2, 3 and 4 are similar (refer to Appendix Section 6 for the TEPI utility function). For scenario 3, the TEPI design does very well in selecting the optimal dose, and all designs consistently select dose level 3 as the optimal dose most frequently. For Scenario 4, the extended mTPI and TEQR designs choose dose level 1 as the optimal dose most frequently. The Eff-Tox design also chooses dose level 1, which has the highest trade-off value calculated per the Eff-Tox method, as the optimal dose most frequently. The TEPI and OBD Isotonic designs choose dose level 2 as the optimal dose more frequently than dose level 1. For the TEPI design, this may be due to the fact that the design allows enrollment of the next cohort at the current dose level 2 even if the toxicity rate is between 0.2 and 0.33 provided the efficacy rate is high enough (refer to TEPI dosing decision table in Appendix Section 6). For the TEPI and OBD Isotonic designs, when we start at dose level 2, they may take a longer time or more subjects to de-escalate given the acceptable efficacy at dose 2. However, both designs eventually select dose level 1 as the optimal dose with higher probability than dose level 2, as the sample sizes increase in our simulations (results not shown here). For scenario 5, all designs pick dose level 3 as the optimal dose most frequently. The OBD Isotonic design performs very well for this scenario (umbrella-shaped dose response curve), while the Eff-Tox and TEPI designs have a lower probability of selecting the optimal dose for such a dose-response curve. In summary, among the designs considered, the extended mTPI design selects the optimal dose more accurately than the other designs for most of the scenarios. The extended TEQR design performs as well as or better than the Eff-Tox design in terms of accuracy of optimal dose selection in most of the scenarios considered.

## 4 Discussion

We have first compared the frequentist TEQR design with the Bayesian mTPI design for accuracy of MTD selection, when using the same stopping rules for both designs. In the scenarios considered, the Bayesian mTPI design is generally more accurate in selecting the true MTD than the frequentist TEQR design, when identical stopping rules and the same or similar sample sizes are used for both the designs. The mTPI design also puts a larger percentage of patients at the MTD and under-doses a smaller percentage of patients compared to the TEQR design. For both designs, given the same cohort size, the accuracy of MTD selection increases when the total sample size is increased.

We then extended the mTPI and TEQR designs to also consider efficacy in addition to safety in dose selection, in a moderately sized trial. In our extended mTPI or TEQR trial designs, isotonic regression is always applied to the observed DLT rates at the end of the trial, since the true DLT rate is always assumed to increase with an increase in dose. The technique that is most appropriate to apply to the observed response rates depends on the drug's properties (Fig. 1). For this, clinical knowledge or judgement about the true underlying response rates of the study drug is required to have a good initial guess at the shape of the true dose-response curve.

When the true underlying response rates are thought to increase monotonically with an increase in dose or are thought to first increase monotonically and then plateau after a certain dose level, isotonic regression can also be applied to the observed response rates at the end of the extended mTPI or TEQR trial. The optimal dose level for safety and efficacy is chosen to be the highest dose level for which the DLT rate after applying isotonic regression is below or at the chosen toxicity threshold (e.g. DLT rate <= 0.33), only if the threshold for response rate is crossed at that dose. If the threshold for response rate is not reached at the highest dose level at which the smoothed DLT rate is below or at the toxicity threshold, then no dose level is chosen as optimal for safety and efficacy (Fig. 3).

When the underlying true response rates are thought to possess a clear peak (e.g. umbrella shaped dose-response curve), isotonic regression on the differences in observed response rates between adjacent dose levels, along with the sign of these differences, can be used to reveal or identify this peak dose level for efficacy. This information of the peak dose level for efficacy can then be used in conjunction with the dose level picked as the highest dose level that is safe, to select an optimal dose for safety and efficacy. For example, if the peak dose level identified for efficacy is equal to or lower than the highest dose level that is safe, then the peak dose level identified for efficacy is chosen as the optimal dose for safety and efficacy, assuming that the peak is above the specified efficacy threshold – if not, no dose level is chosen as the optimal dose. If the peak dose level identified for efficacy is higher than the highest dose level that is safe, then the highest dose that is safe is chosen as the optimal dose, only if the response rate at that dose is greater than or equal to the efficacy threshold – if not, no dose is chosen as the optimal dose. Thus, we cannot select a dose that exceeds the threshold toxicity, but if the maximum/peak efficacy of the drug is reached at a lower dose, we can select that dose as optimal assuming the efficacy threshold is crossed at that dose (Fig. 2, Fig. 3).

When we use isotonic regression on the differences in observed response rates between adjacent dose levels when there is no peak in the true response rates (for e.g. monotonically increasing true response rates), we find that no dose level is selected as the peak very frequently. For a plateauing response curve, we find that no dose level is selected as the peak quite frequently and the dose level at which the response rate starts plateauing is not chosen frequently as the peak dose. Thus, the plateau/peak is not clearly revealed by this technique. Hence, in these cases (monotonically increasing and plateauing response rates), applying isotonic regression on the response rates themselves provides better performance than applying isotonic regression to the differences in observed response rates between adjacent dose levels.

We compared the extended mTPI and TEQR designs to the Eff-Tox design, the OBD Isotonic design and the TEPI design for accuracy of optimal dose selection for some scenarios of true efficacy and toxicity rates. We found that the extended mTPI design selects the optimal dose more accurately than the other designs for most of the scenarios considered. The extended TEQR design performs as well as or better than the Eff-Tox design in terms of the accuracy of optimal dose selection for most of the scenarios considered.

### 4.1 Conclusion

In summary, we have proposed two designs that incorporate toxicity and efficacy in dose selection, and found that the extended mTPI design selects the optimal dose more accurately than the other designs for most of the scenarios. We found that isotonic regression itself applied on the **differences** in observed response rates between adjacent dose levels could be used to identify the peak of a dose-response curve with a clear maximum, such as a convex umbrella-shaped dose-response curve. For other dose-response curves, such as monotonically increasing or plateau, applying isotonic regression to both the observed DLT and response rates independently can be used to determine the optimal dose for toxicity and efficacy. Finally, we note that our models and isotonic regression method to identify an optimal dose for safety and efficacy can be used for other binary efficacy endpoints, such as the progression-free survival or overall survival at a landmark time (e.g, 3 months).

## Figures and Tables

**Appendix Table 1:** Parameters for the mTPI and TEQR Designs.

Parameter	mTPI Design	TEQR Design
Number of simulations	1000	1000
Target toxicity probability p_T_	0.2	0.2
ε_1_	0.05	0.05
ε_2_	0.05	0.05
Starting dose	Dose level 2	Dose level 2
DLT probability deemed to be too toxic to allow further study at that dose level	If Pr (*p*_*i*+1_ > *p*_*T*_|data) > *0.95, then treat next cohort of patients at dose i (see Ji* et al.*, 2010). If the posterior probability that DLT occurs at dose i+1 is greater than the target DLT rate given the data is greater than 0.95, then treat the next cohort of patients at the same dose and exclude doses i+1 and higher from the trial for evaluation.*	0.34
Planned sample size at MTD^a^	NA	50
Maximum number of cohorts^a^	NA	30
True DLT rate at each dose level	Values from Table 1	Values from Table 1
True Response rate at each dose level	Values from Table 1	Values from Table 1

We start from Dose Level 2 to allow for immediate de-escalation to dose level 1, if required.

a Maximum sample size for the mTPI design in most of our simulations is 50, the cohort size is 5 and the maximum number of cohorts is 10.

**Appendix Table 2:** Results for Dose Selection for the Extended TEQR Design for a Case of Non-Zero Correlation between Toxicity and Efficacy.

Extended TEQR Design			
Dose Level	% of Times Dose Level is Chosen for Toxicity	% of Times Dose Level is Chosen for Efficacy	% of Times Dose Level is Chosen as Optimal for Toxicity and Efficacy
No dose is chosen	0%	5%	28%^a^
1	0.8%	0%	0%
2	3.2%	28.4%	0.6%
3	31.8%	29.7%	17.2%
4	63.7%	29.0%	53.7%
5	0.5%	7.9%	0.5%

a These results for the % of times each dose level is selected as optimal for toxicity and efficacy are based on simulations, and are not calculations based on the % of times (or probability) that each dose level is chosen for toxicity and for efficacy, since the correlation coefficient r is not 0 in this example.

**Appendix Table 3a:** Results for Accuracy of Dose Selection for the Extended TEQR Design for Monotonically Increasing True DLT and Response Rates Using the Differences in Observed Response Rates between Adjacent Dose Levels

Extended TEQR Design		
Dose Level	% of Times Dose Level is Chosen for Toxicity	% of Times Dose Level is a Peak for Efficacy
No dose level is chosen	0%	44.2%
1	0.8%	0.1%
2	3.2%	12%
3	31.8%	22.9%
4	63.7%	12.8%
5	0.5%	7.9%
6	0%	0.1%

**Appendix Table 3b:** Results for Accuracy of Dose Selection for the Extended TEQR Design for Monotonically Increasing True DLT and Response Rates Using the Differences in Observed Response Rates between Adjacent Dose Levels.

Extended TEQR Design		
Dose Level	% of Times Dose Level is Chosen for Toxicity	% of Times Dose Level is a Peak for Efficacy
No dose level is chosen	0%	53.0%
1	0.8%	0.1%
2	3.2%	8.2%
3	31.8%	15.5%
4	63.7%	15.2%
5	0.5%	7.9%
6	0%	0.1%

**Appendix Table 4a:** Results for Accuracy of Dose Selection for the Extended TEQR Design for Monotonically Increasing True DLT Rates and Plateauing Response Rates Using the Differences in Observed Response Rates between Adjacent Dose Levels

Extended TEQR Design		
Dose Level	% of Times Dose Level is Chosen for Toxicity	% of Times Dose Level is a Peak for Efficacy
No dose level is chosen	0%	36.2%
1	0.8%	0.1%
2	3.2%	13.5%
3	31.8%	24.7%
4	63.7%	18.3%
5	0.5%	7.0%
6	0%	0.2%

**Appendix Table 4b:** Results for Accuracy of Dose Selection for the Extended TEQR Design for Monotonically Increasing True DLT Rates and Plateauing Response Rates Using the Differences in Observed Response Rates between Adjacent Dose Levels

Extended TEQR Design		
Dose Level	% of Times Dose Level is Chosen for Toxicity	% of Times Dose Level is a Peak for Efficacy
No dose level is chosen	0%	31.8%
1	0.8%	0%
2	3.2%	11.7%
3	31.8%	34.2%
4	63.7%	13.6%
5	0.5%	8.7%

**Appendix Table 5a:** Results for Accuracy of Dose Selection for the Extended TEQR Design for Monotonically Increasing True DLT Rates and Umbrella-Shaped Response Rates Using the Differences in Observed Response Rates between Adjacent Dose Levels

Extended TEQR Design		
Dose Level	% of Times Dose Level is Chosen for Toxicity	% of Times Dose Level is a Peak for Efficacy
No dose level is chosen	0%	22.7%
1	0.8%	0.1%
2	3.2%	15.5%
3	31.8%	32.0%
4	63.7%	27.6%
5	0.5%	2.1%

**Appendix Table 5b:** Results for Accuracy of Dose Selection for the Extended TEQR Design for Monotonically Increasing True DLT Rates and Umbrella-Shaped Response Rates Using the Differences in Observed Response Rates between Adjacent Dose Levels

Extended TEQR Design			
Dose Level	% of Times Dose Level is Chosen for Toxicity	% of Times Dose Level is a Peak for Efficacy	% of Times Dose Level is Selected as Optimal for Safety and Efficacy
No dose level is chosen	0%	15.0%	32%
1	0.8%	0.3%	0%
2	3.2%	1.7%	1.4%
3	31.8%	68.7%	63.4%
4	63.7%	9.4%	3.1%
5	0.5%	4.9%	0.1%

**Table:** Probability of Toxicity and Efficacy Limits for Dose Acceptability Rules.

Parameter	Value
Prob (tox) upper limit (π_T_*)	0.33000
Lower prob cutoff for prob of toxicity (*p*_T,L_)	0.10000
Prob (eff) lower limit (π_E_*)	0.40000
Lower prob cutoff for prob of efficacy (*p*_E,L_)	0.10000

**Table:** Trade-off Function Elicited Points (3 points to define the trade-off function contour).

	π_E_	π_T_
**(π**_1,E_*, 0)	0.50000	0.00000
**(1, π**_2,T_*)	1.00000	0.65000
**(π**_3,E_**, π**_3,T_**)**	0.70000	0.25000

**Table:** Elicited Means (Prior Toxicity, Prior Efficacy).

Dose	Toxicity	Efficacy
1	0.0200	0.2000
2	0.0400	0.4000
3	0.0600	0.6000
4	0.0800	0.8000
5	0.1000	0.9000
6	0.1500	0.9500

**Table:** Decision Table for TEPI Design.

	Efficacy rate between 0 and 0.4	Efficacy rate between 0.4 and 0.7	Efficacy rate between 0.7 and 0.9	Efficacy rate between 0.9 and 1
Toxicity rate between 0 and 0.1	E	E	E	E
Toxicity between 0.1 and 0.2	E	E	E	S
Toxicity between 0.2 and 0.33	D	S	S	S
Toxicity between 0.33 and 1	D	D	D	D

E, D and S denote "escalation", "de-escalation", and "stay" respectively.

**Figure:**
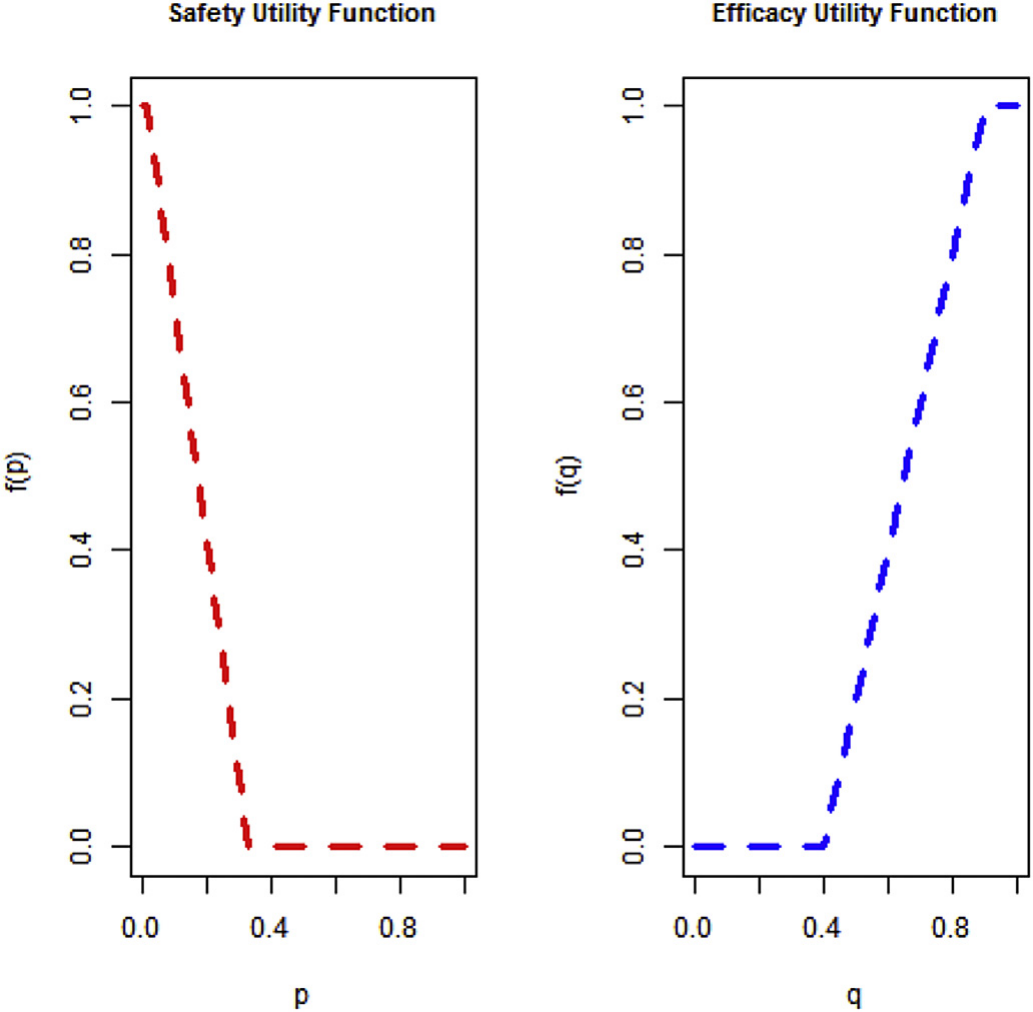


## Appendix

### 2. Results for Dose Selection for the Extended TEQR Design with a Non-Zero Correlation Coefficient between the True Toxicity and Efficacy Rates

The monotonically increasing true DLT and response rates with an increase in dose shown in Table 1 are used in the simulations and isotonic regression is applied independently to the observed DLT rates and to the observed response rates at the end of each simulated trial.

We use the usual stopping rules of the TEQR design, namely stop the trial when the planned MTD sample size is reached or when dose level 1 is too toxic. The results in Appendix Table 2 are based on a sample size at the MTD of 50, a cohort size of 5 and a non-zero correlation coefficient of r equal to 0.22.

We choose dose level 4 as the optimal dose for safety and efficacy most frequently in this case.

The results for optimal dose selection are similar to those obtained for the “no correlation between the efficacy and toxicity rates” case i.e. r = 0 case, with the other input parameters and stopping rules remaining the same.

### 3. Incorporating Safety and Efficacy: True DLT and Response Rates that Increase Monotonically with an Increase in Dose

We use the usual stopping rules of the TEQR design, namely stop the trial when the planned MTD sample size is reached or when dose level 1 is too toxic. The results in Appendix Table 3 a and b are based on a sample size at the MTD of 50, a cohort size of 5 and a correlation coefficient r = 0.

The monotonically increasing true DLT rates shown in Table 1 are used to produce the simulations in Appendix Table 3 a and b. The monotonically increasing response rates with an increase in dose shown in Table 1 are used in the simulations in Appendix Table 3a. Isotonic regression is applied to the observed DLT rates and isotonic regression is applied to the differences in observed response rates between adjacent dose levels at the end of each simulated trial (Appendix Table 3 a and b).

We calculate the differences in the observed response rates between adjacent dose levels at the end of each simulated trial i.e. between dose level 1 and 2, 2 and 3, 3 and 4 etc. We then investigate where the differences in observed response rates between adjacent dose levels switch from a negative sign to a positive sign after applying isotonic regression to these differences, to determine whether there is a peak at a certain dose level in the observed response rates. If in a simulation the difference in response rates between dose levels 2 and 3 is negative and that between dose levels 3 and 4 is positive, then dose level 3, is considered the peak. If the difference in response rates between dose levels 1 and 2 is negative, that between dose levels 2 and 3 is zero and that between dose levels 3 and 4 is positive, then dose level 3, is the peak. If all the differences between adjacent dose levels are negative in a simulation, then no dose level chosen as the peak for that simulation. If all the differences between adjacent dose levels are positive in a simulation, then dose level 1 is the peak.

In this example, dose level 3 is what is picked most frequently as the peak but it is selected only 23% of the time. Also, note that no dose level is chosen as the peak dose level for efficacy 44% of the time. Thus, there is no peak in the observed response rates at dose level 3. Based on the true response rates in Table 1, the differences in true response rates between adjacent dose levels are always negative and there is no peak at dose level 3. Hence, the results in Appendix Table 3a reflect this underlying scenario.

As another example, if the true response rate at dose level 1, 2, 3, 4, 5 and 6 are 0.1, 0.2, 0.3, 0.4, 0.5 and 0.6 respectively i.e. the difference in true response rates between adjacent dose levels is always −0.1, and isotonic regression is applied to the observed differences at the end of each of the 1000 simulations, the results in Appendix Table 3b are obtained. It is seen, in this scenario, that dose level 3 is picked as the peak most frequently, but only 16% of the time, and that no dose level is selected as the peak 53% of the time, reflecting the underlying scenario in true response rates where there is no peak.

Hence, in general, when the true response rates are monotonically increasing with an increase in dose, applying isotonic regression on the differences in response rates between adjacent dose levels will show that no dose level is selected as the peak dose most frequently; thus, in this case, applying isotonic regression on the observed response rates themselves can help determine the dose to be chosen for efficacy.

### 4. Incorporating Safety and Efficacy: Plateauing True Response Rates with an Increase in Dose

We use the usual stopping rules of the TEQR design, namely stop the trial when the planed MTD sample size is reached or when dose level 1 is too toxic. The results in Appendix Table 4a and b are based on a sample size at the MTD of 50, a cohort size of 5 and a correlation coefficient r = 0.

The monotonically increasing true DLT rates shown in Table 1 are used to produce the simulations in Appendix Table 4a and 4b. The plateauing true response rates with an increase in dose shown in Table 1 are used in the simulations in Appendix Table 4a. Isotonic regression is applied to the observed DLT rates and isotonic regression is applied to the differences in observed response rates between adjacent dose levels at the end of each simulated trial (Appendix Table 4a and 4b).

We calculate the differences in the observed response rates between adjacent dose levels at the end of each simulated trial and apply isotonic regression to these differences. We then investigate where these differences switch signs after isotonic regression, to help determine whether there is a peak at a certain dose level in the observed response rates. In this example, there is no peak 36% of the time, dose level 3 is chosen as the peak most frequently, and dose level 4 is chosen as the peak only 18% of the time. Thus, there is no clear peak or plateau in response rates at dose level 4. Based on the true underlying response rates in Table 1, there is a plateau from dose level 4. However, the results in Appendix Table 4a do not clearly show that there is a peak or plateau in response rates at dose level 4.

As another example, if the true response rate at dose level 1, 2, 3, 4, 5 and 6 are 0.1, 0.35, 0.5, 0,5, 0.5 and 0.5 respectively, and isotonic regression is applied to the observed differences at the end of each of the 1000 simulations, the results in Appendix Table 4b show that dose level 3, the dose level that is selected as the peak most frequently, is selected only 34% of the time. However, no dose level is selected as the peak almost as frequently, at 32% of the time. Thus, the results in Appendix Table 4b do not clearly show that there is a peak or plateau in response rates at dose level 3, as present in the true underlying response rates.

Hence, applying isotonic regression on the differences in observed response rates between adjacent dose levels in the case the response rates plateau with an increase in dose will show that no dose level is selected as the peak dose frequently and also does not reveal the plateau/peak clearly; thus, in this case, when there is no clear peak, applying isotonic regression on the observed response rates themselves can help determine the dose to be chosen for efficacy.

### 5. Incorporating Safety and Efficacy: Response Rates that Follow an Umbrella-Shaped Dose-Response Curve

We use the usual stopping rules of the TEQR design, namely stop the trial when the planned MTD sample size is reached or when dose level 1 is too toxic. The results in Appendix Table 5a and b are based on a sample size at the MTD of 50, a cohort size of 5 and a correlation coefficient r = 0.

The monotonically increasing true DLT rates with an increase in dose shown in Table 1 are used to produce the simulations in Appendix Table 5a and b. The umbrella-shaped true response rates of 0.1, 0.3, 0,4, 0.45, 0.2 and 0.05 at dose levels 1, 2, 3, 4 and 5 respectively, where the response rate peaks at dose level 4, are used in the simulations in Appendix Table 5a. Isotonic regression is applied to the observed DLT rates and isotonic regression is applied to the differences in observed response rates between adjacent dose levels at the end of each simulated trial (Appendix Table 5a and b).

We calculate the differences in the observed response rates between adjacent dose levels at the end of each simulated trial and apply isotonic regression to these differences. We then investigate where these differences switch signs after isotonic regression, to help determine whether there is a peak at a certain dose level in the observed response rates. In this example, dose level 3 is chosen slightly more frequently than dose level 4 as the peak. Based on the true underlying response rates in Table 1, there is a peak at dose level 4. Thus, the results in Appendix Table 5a do not reflect this peak at dose level 4 as clearly. It is seen from the example below and in the main text (Table 3) that when the peak in response rates is at a lower dose level, such as at dose level 3, it is revealed clearly by this method. In other words, not as many patients are dosed at the higher dose levels compared to at the lower doses, and it is difficult to reveal the peak in response rates when it is at these higher dose levels.

As an example, if the true response rate at dose level 1, 2, 3, 4, 5 and 6 are 0.05, 0.1, 0.45, 0,3, 0.2, 0.05 respectively, and isotonic regression is applied to the observed differences at the end of each of the 1000 simulations, the results in Appendix Table 5b show that dose level 3 is chosen as the peak most frequently, consistent with the peak at dose level 3 in the true underlying response rates. Dose level 3 is selected as the optimal dose 63% of the time.

Thus, if the true underlying response rates have a clear peak at a certain dose level (umbrella-shaped dose-response curve), isotonic regression on the differences in observed response rates between adjacent dose levels, along with the sign of these differences, can be used to reveal this peak, and determine an optimal dose for toxicity and efficacy (Fig. 2, Fig. 3). The technique works well when the peak in response rates is at a lower dose level rather than a higher dose level where few patients may be dosed.

### 6. Input Parameters Used in Simulations for the Eff-Tox, OBD Isotonic, and TEPI Designs

*Input parameters Used in the Eff-Tox Package for the Eff-Tox Design*.

Cohort size = 5.

Maximum number of subjects = 50.

Starting dose = 2.

Number of simulations = 10000.

The true DLT and response rates at each dose level are as given in Table 4.

*Input parameters Used in the OBD Isotonic Design*.

Cohort size = 5.

Number of cohorts = 10.

Starting dose = 2.

phi = upper bound of toxicity rate = 0.33.

ct = threshold for posterior probability of toxicity (any dose with toxicity probability larger than ct is excluded from the admissible set of doses) = 0.9.

Number of simulations = 10000.

The true DLT and response rates at each dose level are as given in Table 4.

The R code given at the following URL was used along with the input parameters given above: http://odin.mdacc.tmc.edu/∼yyuan/Software/TargetAgent/targetAgentDF.r.

However, this R code was modified to accommodate any starting dose of the simulated trial (for e.g. starting dose = 2). The parameters in the prior beta distribution for toxicity response probability were also changed to reflect the values of ct (=0.9) and phi (=0.33) used in our simulations.

*Input parameters Used in the TEPI Design.*

Number of simulation runs = 1000.

Maximum number of subjects = 50.

Cohort size = 5.

Starting dose = 2.

pT = 0.33 # the maximum accepted toxicity probability.

pE = 0.4 # the minimum accepted efficacy probability.

The true DLT and response rates at each dose level are as given in Table 4.

## Appendix A Supplementary data

mmc1 mmc1

## Footnotes

1 In each set of simulations, only one of the stopping rules is used i.e. either stopping rule (a) is used or (b) is used (not both).
